# Reactivating fear memory under propranolol resets pre-trauma levels of dendritic spines in basolateral amygdala but not dorsal hippocampus neurons

**DOI:** 10.3389/fnbeh.2013.00211

**Published:** 2013-12-23

**Authors:** Gisella Vetere, Valentina Piserchia, Antonella Borreca, Giovanni Novembre, Massimiliano Aceti, Martine Ammassari-Teule

**Affiliations:** ^1^IRCCS Santa Lucia FoundationRome, Italy; ^2^Institute of Cell Biology and Neurobiology, National Research Council (CNR)Rome, Italy

**Keywords:** fear memory reactivation, amygdala, hippocampus, propranolol, dendritic spines, p-ERK

## Abstract

Fear memory enhances connectivity in cortical and limbic circuits but whether treatments disrupting fear reset connectivity to pre-trauma level is unknown. Here we report that C56BL/6J mice exposed to a tone-shock association in context A (conditioning), and briefly re-exposed to the same tone-shock association in context B (reactivation), exhibit strong freezing to the tone alone delivered 48 h later in context B (long term fear memory). This intense fear response is associated with a massive increase in dendritic spines and phospho-Erk (p-ERK) signaling in basolateral amygdala (BLA) but neurons. We then show that propranolol (a central/peripheral β-adrenergic receptor blocker) administered before, but not after, the reactivation trial attenuates long term fear memory assessed drug free 48 h later, and completely prevents the increase in spines and p-ERK signaling in BLA neurons. An increase in spines, but not of p-ERK, was also detected in the dorsal hippocampus (DH) of the conditioned mice. DH spines, however, were unaffected by propranolol suggesting their independence from the ERK/β-ARs cascade. We conclude that propranolol selectively blocks dendritic spines and p-ERK signaling enhancement in the BLA; its effect on fear memory is, however, less pronounced suggesting that the persistence of spines at other brain sites decreases the sensitivity of the fear memory trace to treatments selectively targeting β ARs in the BLA.

## Introduction

Traumatic experience durably impacts on neural connectivity. In humans, comparisons of EEG signal coherence in adult individuals reporting childhood, adulthood, or no history of traumatic experience points to a positive correlation between connectivity over frontal, central, temporal and parietal areas and intensity of the trauma (Cook et al., 2009). Connectivity imaging studies also reveal that patients affected by Post Traumatic Stress Disorder (PTSD) show enhanced wiring in emotion-related regions including the amygdala and the insula (Liberzon and Sripada, 2008; Sripada et al., 2012). In rodents, tone fear conditioning (TFC) drives structural alterations in basolateral amygdala (BLA) neurons consistent with an enhancement in the strength and in the number of synapses. These include an enlargement of postsynaptic spine densities, movements of the polymerization-regulatory protein profilin to dendritic spines (Lamprecht et al., 2006), increased number of spinophilin-immunoreactive spines (Radley et al., 2006), a greater ratio of postsynaptic density (PSD) area to docked vesicles at synapses (Ostroff et al., 2010, 2012) and a net increase in spine density (Heinrichs et al., 2013). Although it is conceivable that connectivity in fear-activated circuits should be reduced upon fear erasure, whether pharmacological treatments reducing fear memory reset physiological levels of connectivity is unknown.

TFC also enhances the activity of the ERK/MAPK pathway in the BLA (Schafe et al., 2000; Di Benedetto et al., 2009). Importantly, the BLA controls adaptation to stress via a β-ARs-mediated regulation of phosphorylated ERK (Grissom and Bhatnagar, 2011). There is evidence that β-ARs antagonists disrupt fear memory (Soeter and Kindt, 2011), but whether they also block fear-induced phospho-Erk (p-ERK) enhancement has not yet been investigated. This point is of importance considering that p-ERK regulates the activity of downstream pathways implicated in the synthesis of proteins involved in synaptic plasticity and experience-dependent structural re-arrangements (Sweatt, 2004).

The present study examines the extent to which β-ARs antagonists, that interfere with fear memory and p-ERK signaling, reinstate pre-trauma levels connectivity in fear-activated neural circuits. We first show that C57BL/6 mice exposed to tone-shock associations in context A and re-exposed to the same association in context B, show intense freezing to the tone alone presented 48 h later in context B. This intense freezing response is accompanied by an increase in dendritic spines and p-ERK signaling in BLA neurons. Administration of the β-ARs blocker propranolol before, but not immediately after, the reactivation test attenuates fear memory assessed drug-free 48 h later but completely prevents the increase in dendritic spines and of p-ERK signaling in BLA neurons. The formation of fear memory also promotes an increase in spines, but not of p-ERK levels, in the dorsal hippocampus (DH) of the conditioned mice. DH connectivity, however, is unaffected by propranolol suggesting that it plays role in the persistence of a consistent level of fear memory.

## Methods and materials

### Animals

C57BL/6J@Ico 8-week old male mice were maintained on a 12 h light/dark cycle with *ad libitum* access to food and water. All experiments were performed in the light cycle and conducted in accordance with the guidelines provided by the European Communities Council Directive of 24 November 1986 (86/609/EEC).

### Behavioral protocol

The behavioral protocol was based on the one designed by Debiec and LeDoux (2004) in which TFC takes place in one context (chamber A), while the reactivation trial and the memory test take place in another context (chamber B).

#### Conditioning

Chamber A was a Plexiglas transparent cage with (28 × 28 × 10 cm) with a squared grid floor illuminated by a 60 W lamp. An ethanol solution (70%) was used to clean the chamber after each mouse underwent the conditioning trial. Chamber B differed from chamber A in shape (trapezoidal shape), color (black walls with a plain gray floor), odor (70% ethanol solution with lemon odor), and light intensity (40 W lamp). Chamber A and B were inserted in an insulated box (TSE Systems GmBH, Germany). From day 1 to 3, mice were handled for 5 min in the experimental room. On day 4, they were introduced in chamber A and, after a 2-minute habituation period, exposed to a 30 s/5 kHz/75 dB tone (conditioned stimulus, CS) co-terminating with a 2 s/1.0 mA foot-shock (unconditioned stimulus, US). Mice were then immediately brought back to their home cage. Pseudo-conditioning consisted in placing mice in chamber A and exposing them to the tone in the absence of foot-shock.

#### Reactivation

On day 5, conditioned mice were injected with saline or propranolol, and pseudo-conditioned mice were injected with saline and immediately after they were submitted to a reactivation trial taking place in chamber B and consisting of a 2-minute habituation period followed by a single CS presentation. To precise the central vs. peripheral effect of propranolol on behavior, a control experiment was run to estimate the effect of pre-reactivation injections of the selective peripheral β-ARs blocker sotalol.

#### Fear memory

Forty-eight hours after the reactivation trial, mice were exposed to four CSs presentations in context B, delivered at a variable inter-tone interval (120 s on average).

#### Behavior recordings

Behavior during conditioning, reactivation trial, and memory test was recorded by means of a video camera mounted 60 cm above the ceiling of chamber A or B, which was connected to a computer equipped with the Ethovision software (Noldus, The Netherlands). The time spent freezing (absence of all but respiratory movements) after CS-US presentation was used to score fear during the conditioning and the retrieval while the time spent freezing during the four CSs presentation was averaged and used as a long term fear memory index (Blanchard and Blanchard, 1969).

### Pharmacological treatments

To keep in line with the Kindt et al. (2009) and Kroes et al. (2010) reports indicating that oral administration of propranolol before retrieval durably alter fear memory in humans, the central/ peripheral βARs blocker (S)-(-)-propranolol hydrochloride (Sigma-Aldrich, St. Louis, MO,USA) was dissolved in saline (0.9%) at a dose of 10 mg/ml and injected intraperitoneally at a volume of 1 ml/kg immediately before the reactivation trial. This dose is also among the highest ones shown to disrupt fear memory in the mouse (Nielson et al., 1999; Cain et al., 2004). In supplemental experiment 1, we injected the selective peripheral βARs blocker (±)-sotalol hydrochloride (Sigma-Aldrich, St. Louis, MO,USA) at the same dose and time-point as propranolol to estimate the respective role of central vs. peripheral βARs blockade on fear memory attenuation. In supplemental experiment 2, we compared the effect of propranolol injected immediately before or immediately after the reactivation trial to assess whether attenuation of fear memory can be ascribed to an effect of the drug on retrieval, on reconsolidation, or both. There is, in fact evidence that post-reactivation injections of the same dose of propranolol is effective in disrupting reconsolidation of spatial memory, inhibitory avoidance and contextual and TFC in the rat (Przybyslawski et al., 1999; Debiec and LeDoux, 2004; Rodriguez-Romaguera et al., 2009; Muravieva and Alberini, 2010).

### Spine density analysis

Ninety minutes after the retention test, mice were deeply anaesthetized with chloral hydrate (400 mg/kg i.p.) and perfused transcardially with 0.9% saline solution (*N* = 7 mice per group). Brains were dissected and immediately immersed in a Golgi-Cox solution at room temperature for 6 days (Glaser and Van der Loos, 1981). On the seventh day, brains were transferred in a 30% sucrose solution for cryoprotection and then sectioned with a vibratome. Coronal sections (100 μm) were collected and stained according to the method described by Gibb and Kolb (1998). Spine density was analyzed on class I spiny neurons which represent the predominant cell type in the basolateral amygdala nuclei (McDonald, 1982). Class I neurons, which have a pyramidal soma, one or two thick apical dendrites and multiple thin basal dendrites, were identified with a light microscope (Leica DMLB) under low magnification (20×/NA 0.5). Three neurons within each hemisphere were taken from each animal. On each neuron, five 30–100 μm dendritic segments of secondary and tertiary branch order of basolateral amygdala dendrites were randomly selected (Leuner et al., 2003) and counted using Neurolucida software. Only protrusions with a clear connection of the head of the spine to the shaft of the dendrite were counted as spines (Horner and Arbuthnott, 1991). Statistical comparisons were made on single neuron values obtained by averaging the number of spines counted on segments of the same neuron. The analysis was conducted by an experimenter blind to the experimental condition. To control for the regional specificity of fear memory-induced structural changes in the hippocampus, CA1 dorsal (*N* = 3) and ventral (*N* = 3) hippocampal neurons were selected within each hemisphere in the same animals, and spine density counts were performed on basal dendrites using to the same quantification method.

### Western blots quantification of p-ERK levels

Punches for western blots were taken 90 min after the memory test from the BLA and the DH of mice experiencing the three conditions (4 mice per group) using a 1 mm punch tool (Fine Science Tools, Foster City, CA) from 1 mm thick sections cut on a frozen brain matrix (ASI Instruments). Punches were homogenized and extracted in RIPA buffer (10 mM Tris-HCl, pH 7.5, 150 mM NaCl, 2% Nonidet P-40, 5 mM EDTA, 0.1 mM phenylmethylsulfonyl fluoride, 1 mM β-glycerophosphate, 1 mM sodium orthovanadate, 10 mM sodium fluoride, 0.1 M SDS, 1% protease inhibitor cocktail-Sigma Aldrich) for 5 min on ice and centrifuged for 10 min at 4°C (8000 rpm). The supernatant was collected, and protein content was quantified by Bradford colorimetric assay (Biorad, Milano, Italy). Homogenates containing 30 μg of total protein were separated by 4–15% gradient sodium dodecyl sulfate–polyacrylamide gels (BIO-RAD Laboratories, Hercules, CA) and transferred to nitrocellulose membranes (BIO-RAD Laboratories, Hercules, CA). Western blots were blocked in 5% non-fat dry milk in TBST buffer (0.1% Tween 20 in Tris–borate saline) then incubated with p-ERK1/2 antibody (1:1000; Immunological Science). The two bands corresponding to p-ERK42 and p-ERK44 consistently varied in the same manner and were thus analyzed together. The normalization of the data was performed by probing membranes with the antibody against the total ERK protein (1:1000; Cell Signaling). Blots were then incubated with appropriate secondary antibody conjugated to horseradish peroxidase (Chemicon) and developed by ECL Western Blotting Analysis System (GE Healthcare). Anti-Tubulin antibody (1:1000; Millipore) was used as a loading control for all experiments. Optical densities of the bands were analyzed using NIH Image software.

### Immunohistochemistry detection of p-ERK positive neurons

Mice were deeply anesthetized with chloral hydrate (40 mg/kg) injected intraperitoneally and then transcardially perfused with a 4% paraformaldehyde solution. Brains were removed from the skull and left overnight in a 4% paraformaldehyde solution. On the next day, brains were transferred in a 30% sucrose solution and left overnight. On the next day, frontal 30 um cryostat slices were sectioned from entire brain and immediately prepared for immunohistochemistry.

The slices were treated with 3% H_2_O_2_ and then washed with phosphate buffer saline. The immunohistochemistry protocol was performed according the manufacturing procedure of ULTRATEK HRP KIT (ScyTek laboratories). Sections for immunohistochemistry were incubated for 72 h at 4°C with primary antibody (pERK immunological science; 1:200) diluted in PBS with 0.3% triton X100. Section were then washed with PBS and incubated with biotinylated goat antirabbit secondary antibody. After washing, sections were incubated with avidin biotynilated peroxidase complex. Peroxidase activity was visualized with 3,3′-diaminobenzidine. For light microscopy immunohistochemistry, slices were mounted on superfrost plis slides, air dried, quickly dehydrated, and coverslipped.

### Statistical analyses

Results were expressed as means ± s.e.m. Between-group differences in freezing behavior, spine density, protein levels were estimated by means of a One-Way ANOVA with group as the main factor. In supplemental experiment 1, differences in freezing behavior during the long term memory test between mice receiving pre-reactivation injections of sotalol or saline were estimated by means of a *t*-test. In Supplemental experiment 2, the effect of pre- vs. post-reactivation injections of propranolol on long term fear memory was estimated by means of a Two-Way ANOVA with treatment (propranolol vs. saline) and injection time (pre-vs. post reactivation) as main factors. *Post-hoc* pair comparisons were carried out where necessary by means of the LSD test.

## Results

### Behavior

**Figure 1:**
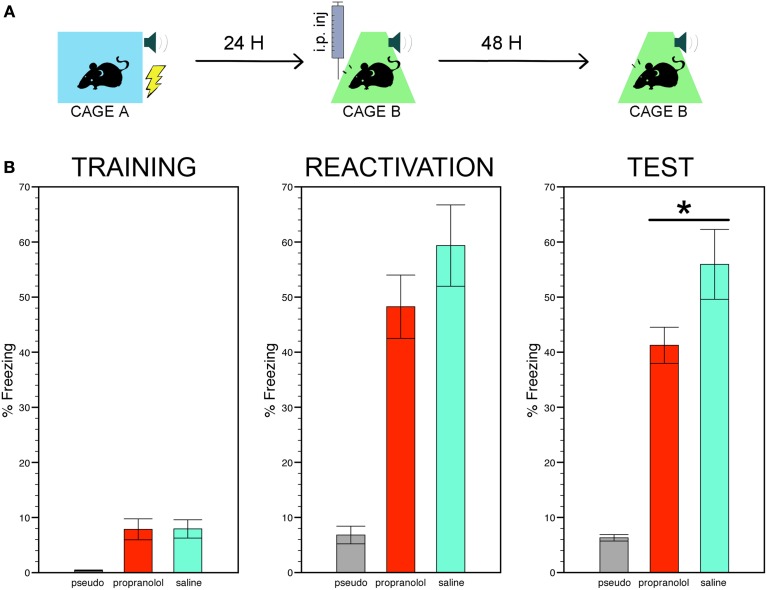
**Reactivating fear under propranolol reduces long term fear memory. (A)** Experimental protocol: mice subjected to tone fear conditioning in context A were then injected with the β-adrenergic receptor blocker propranolol or with saline before their re-exposure to the same tone-shock association in context B. Fear memory was assessed 48 h later by monitoring freezing to the tone alone in context B. **(B)** The trained mice injected with propranolol or saline exhibited stronger freezing during the conditioning, the reactivation trial and the memory test compared to the pseudo-trained non-shocked mice injected with saline (7 mice per group). Among the trained mice, those receiving pre-reactivation injections of propranolol showed significantly less freezing during the memory test than those receiving saline injections. Data are expressed as mean ± s.e.m. ^*^p < 0.05.

Figure 1 shows a cartoon depicting the three phases of the behavioral protocol (Figure 1A) and the freezing scores of the three experimental groups during the conditioning, the reactivation trial, and the memory test (Figure 1B).

#### Conditioning

The ANOVA performed on the time mice spent in freezing in chamber A during the entire duration of the conditioning trial, i.e., before and during the CS was paired with the US or, for the pseudo-conditioned mice, before and during the CS was not followed by the US, revealed a significant effect of group [*F*_(2, 31)_ = 6.22, *p* < 0.01]. *Post-hoc* comparisons then showed that two trained groups exhibited more freezing than the pseudo-trained group (*p* < 0.005 for both comparisons).

#### Reactivation

The reactivation trial was run by delivering the CS in context B instead of context A. As for the conditioning, significant differences between the freezing scores of the three experimental groups were found during the reactivation trial [effect of group, *F*_(2, 31)_ = 18.83, *p* < 0.001]. *Post-hoc* comparisons between the two trained groups revealed that although mice injected with propranolol showed less freezing that their saline-injected counterpart, these scores did not differ significantly. The scores of these two groups were, however, significantly higher than those of the pseudo-trained mice (*p* < 0.001 for each pair comparison). Pre-reactivation injections of sotalol did not affect freezing during the reactivation trial (sotalol- vs. saline-injected trained mice, *t* = 0.67; *p* = 0.51). In Supplemental experiment 2, the effect of pre- vs. post-reactivation injections of propranolol on long term fear memory was estimated by means of a Two-Way ANOVA with treatment (propranolol vs. saline) and injection time (pre-vs. post reactivation) as main factors. *Post-hoc* pair comparisons were carried out where necessary by means of the LSD test.

#### Fear memory

The time spent freezing in chamber B 48 h after the reactivation trial was then taken as an index of long term fear memory. Statistical comparisons of the freezing scores still showed significant differences between the three experimental groups [*F*_(2, 31)_ = 28.77, *p* < 0.001] with the trained mice receiving pre-reactivation injections of propranolol or saline still exhibiting significantly more freezing than the pseudo-trained mice (*p* < 0.001 for each pair comparison). However, among the trained mice, those receiving propranolol showed significantly less freezing than those receiving saline (*p* < 0.05). Pre-reactivation injections of sotalol did not decrease freezing during the memory test (sotalol- vs. saline-injected mice, *t* = 0.63; *p* = 0.53, NS, Figure S1 thus indicating that propranolol disrupts fear memory via selective blockade of central β ARs. Differently from data obtained in the rat (Przybyslawski et al., 1999; Debiec and LeDoux, 2004; Rodriguez-Romaguera et al., 2009; Muravieva and Alberini, 2010), the comparison of pre-vs. post-reactivation injections of propranolol on long term fear memory revealed that propranolol injected immediately after the reactivation trial was ineffective in decreasing freezing in the conditioned mice Figure S2). A significant treatment × injection time interaction [*F*_(1, 16)_ = 6.33, *p* < 0.05] was found with *post-hoc* comparison indicating (i) a significant reduction of freezing in pre- vs. post-reactivation propranolol injected mice (*p* < 0.05), and (ii) equivalent levels of freezing in post-reactivation propranolol- vs. saline-injected mice (*p* = 0.10). These findings therefore suggest that the effect of the drug on long term fear memory is unlikely to be ascribed to a disruption of memory reconsolidation.

### Dendritic spines

#### Basolateral amygdala

**Figure 2:**
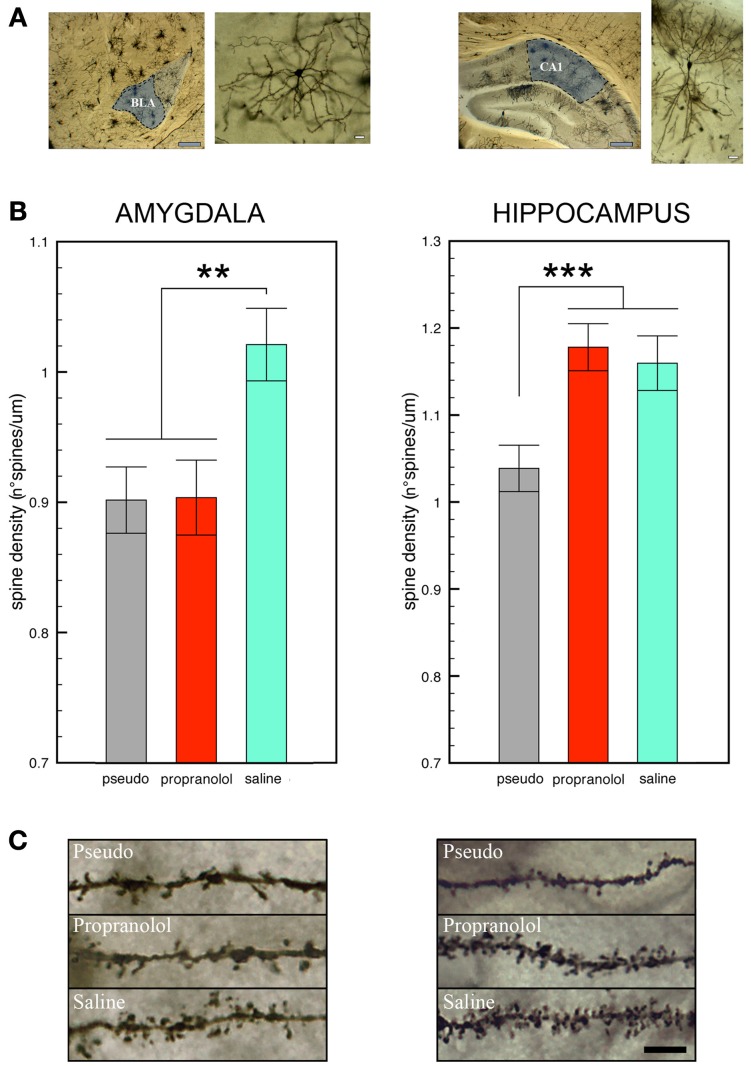
**Propranolol prevents the increase in spine density observed in basolateral amygdala, but not in CA1 dorsal hippocampus, neurons upon fear memory testing. (A)** Representative Golgi-Cox stained neurons in the basolateral amygdala (BLA, left panel) and the CA1 field of the dorsal hippocampus (DH, right panel) Gray scale bars: 250 μm; white scale bars: 10 μm. **(B)** Mice subjected to tone fear conditioning and injected with saline before the reactivation trial showed an increase in dendritic spines on BLA (left panel) and CA1 DH (right panel) neurons upon the memory test in comparison with saline-injected pseudo-trained mice. Pre-reactivation injections of propranolol blocked the increase in spines in BLA but not in CA1 DH neurons. Data are expressed as mean ± s.e.m. ^**^p < 0.01; ^***^p < 0.005 **(C)** Representative photomicrographs showing Golgi-stained BLA (left panel) and CA1 DH (right panel) neuron segments in each experimental condition. Black scale bar: 5 μm.

Statistical comparison of the number of spines counted on BLA neurons revealed a significant effect of group [*F*_(2, 139)_ = 6.27, *p* < 0.01]. *Post-hoc* analyses then showed that spine density was significantly higher in the trained mice receiving pre-reactivation injections of saline in comparison with those receiving propranolol (*p* < 0.005) or with the pseudo-trained (*p* < 0.01) mice (Figure 2). Importantly, no difference in spine density was found between the two latter groups (propranolol-injected trained mice vs. saline-injected pseudo-trained mice, *p* = 0.96 NS) indicating that the treatment fully reset spine density to the level of the pseudo-trained mice.

#### Dorsal hippocampus

A significant effect of group [*F*_(2, 57)_ = 9.05, *p* < 0.001] was also found for the spines counted in DH CA1 pyramidal neurons (Figure 2). Spine density, however, was significantly higher in the trained mice whether they received saline or propranolol, than in the pseudo-trained mice (*p* < 0.001 for both comparisons). In the trained mice, spine density did not significantly differ between those injected with saline or propranolol (*p* = 0.90, NS) therefore revealing that DH spines were unaffected by the treatment. Statistical comparison of spine density in the ventral hippocampus (Figure S3) did not reveal any significant difference [*F*_(2, 57)_ = 0.15, *p* = 0.86, NS].

### Western blots quantification of p-ERK levels

**Figure 3:**
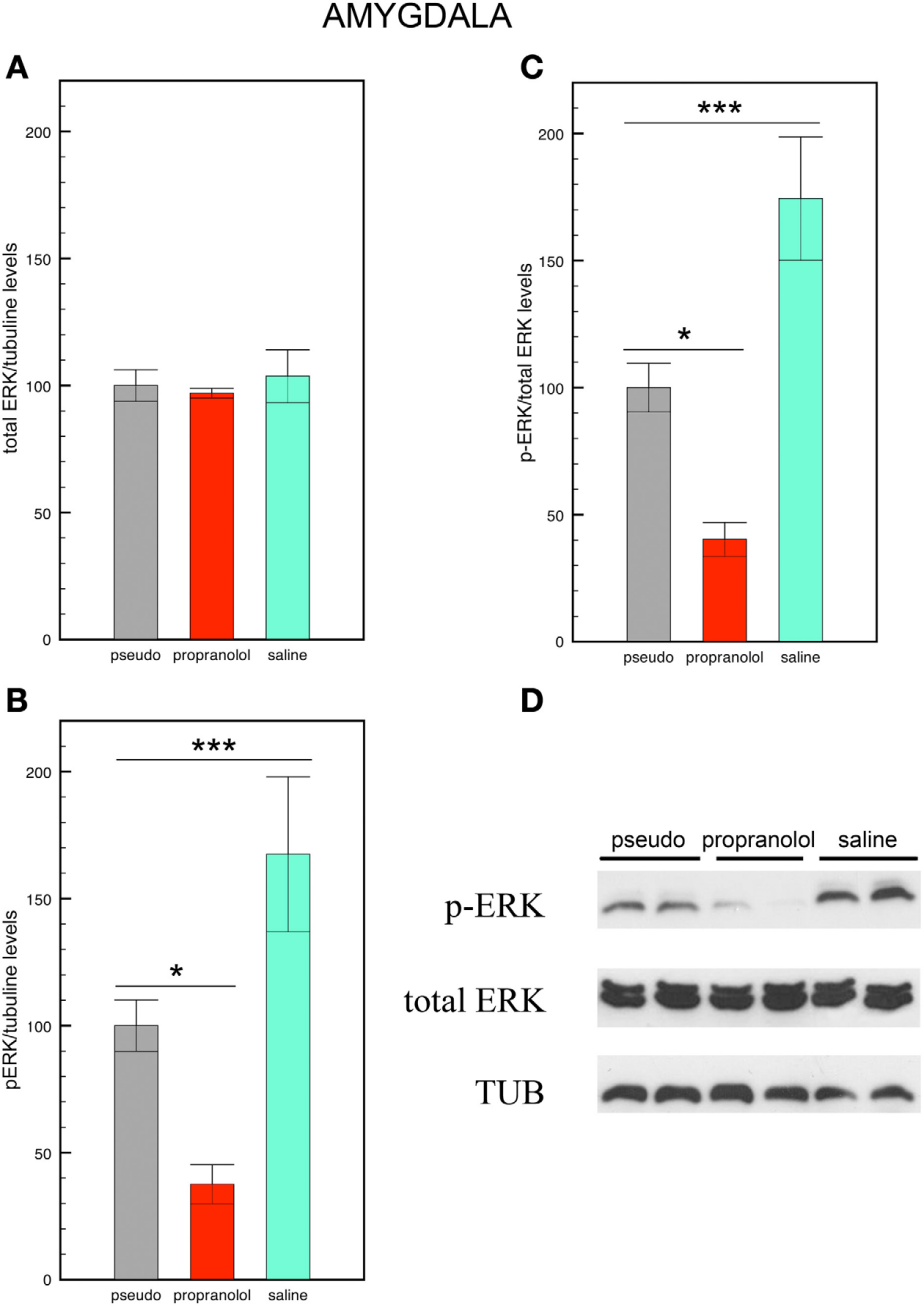
**Propranolol prevents the increase in p-ERK signaling observed in basolateral amygdala upon fear memory testing. (A)** Total ERK levels did not vary across experimental conditions. Mice trained for tone fear conditioning and injected with saline before the reactivation trial showed an increase in p-ERK levels **(B)** and a higher p-ERK/total ERK ratio **(C)** in the basolateral amygdala upon fear memory testing in comparison with saline-injected pseudo-trained mice. Injections of propranolol blocked the increase in p-ERK and p-ERK/total ERK ratio in this region. Data are expressed as mean ± s.e.m. ^*^p < 0.05; ^***^p < 0.005. **(D)** Representative immuno-blotting and densitometry of p-ERK, total ERK, and tubulin expression in basolateral amygdala.

**Figure 4:**
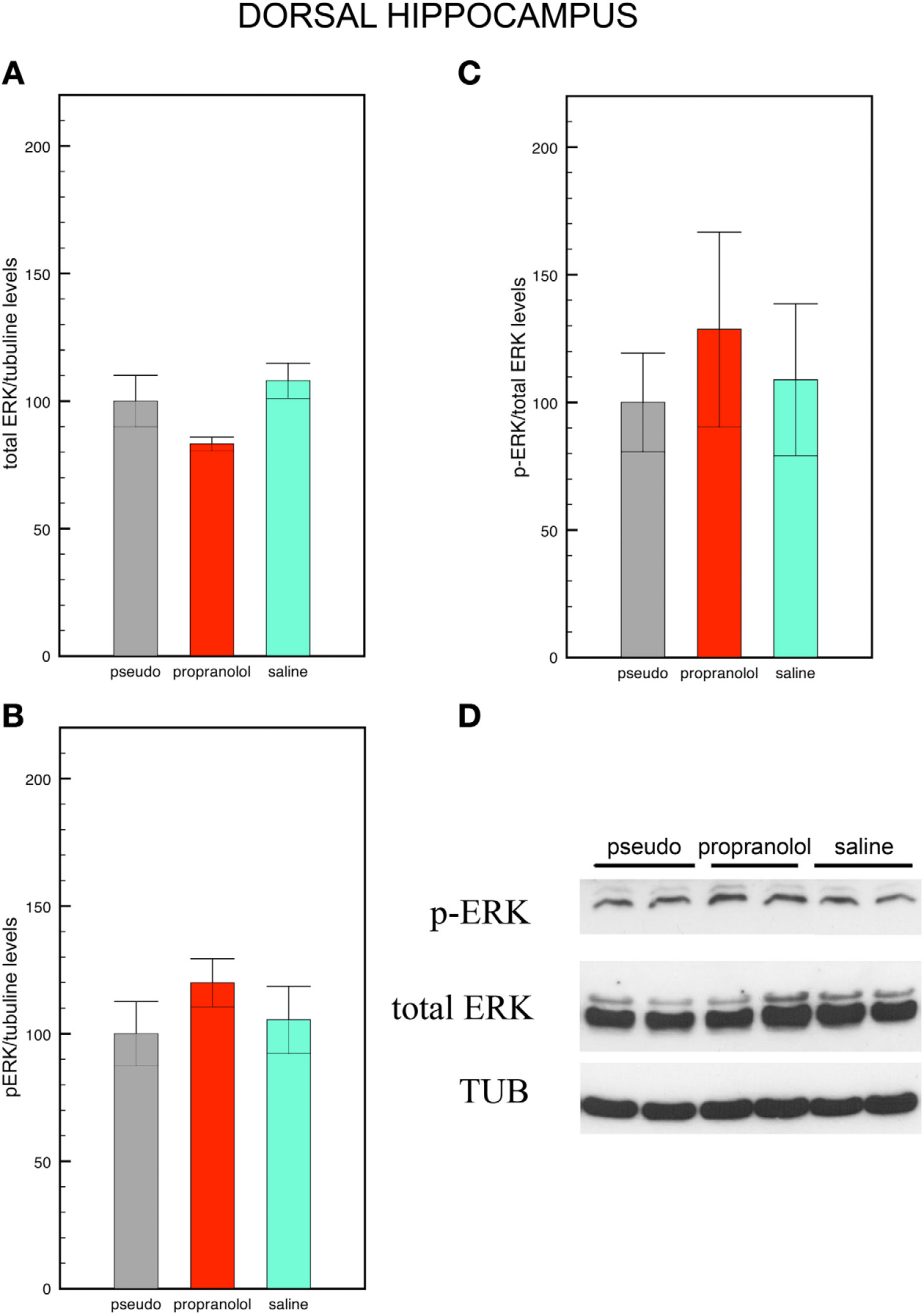
**Fear memory does not modify p-ERK signaling in the dorsal hippocampus**. Mice trained for tone fear conditioning and injected with saline before the reactivation trial did not show any significant difference in total ERK levels **(A)**, p-ERK levels **(B)** and in p-ERK/total ERK ratio **(C)** in the CA1 dorsal hippocampus upon fear memory testing in comparison with pseudo-trained mice. Pre-reactivation injections of propranolol did not modify p-ERK, total ERK, and the p-ERK/total ERK ratio in this region. Data are expressed as mean ± s.e.m. **(D)** Representative immuno-blotting and densitometry of p-ERK, total ERK, and tubulin expression in CA1 dorsal hippocampus.

Western blots were carried out in amygdala and hippocampus homogenates collected 90 min after the memory test and normalized for tubulin levels. Data are shown in Figures 3, 4.

#### Basolateral amygdala

Statistical comparisons of total ERK levels in the amygdala revealed no significant differences between the 3 groups [*F*_(2, 12)_ = 0.13, *p* = 0.88 NS; Figure 3A] In contrast, statistical comparisons of p-ERK [*F*_(2, 12)_ = 17.54, *p* < 0.005; Figure 3B], and of p-ERK/total ERK ratios revealed a significant effect of group [*F*_(2, 12)_ = 14.33, *p* < 0.005; Figures 3C,D]. Pair comparisons then indicated that the p-ERK/total ERK ratio was increased in saline-injected trained mice but decreased in propranolol-injected mice (*p* < 0.05 for both comparisons) relative to saline-injected pseudo-trained mice, thus revealing that fear memory enhances p-ERK signaling in BLA neurons, and that propranolol does not only block this enhancement but reduces p-ERK signaling below the levels observed in saline-injected non-shocked mice.

#### Hippocampus

Statistical comparisons of total ERK levels in the hippocampus also revealed no significant effect of group [*F*_(2, 15)_ = 3.04, *p* = 0.08; Figure4A]. In this region, however, p-ERK levels [*F*_(2, 15)_ = 0.76, *p* = 0.49 NS; Figure 4B] and p-ERK/total ERK ratios did no differ between groups [*F*_(2, 15)_ = 0.23, *p* = 0.79 NS; Figures 4C,D].

### Immunohistochemistry detection of p-ERK positive neurons

Images of p-ERK stained neurons in BLA and CA1 were taken from mice subjected to each training x treatment condition (Figure S4).

#### Basolateral amygdala

p-ERK stained neurons in BLA appeared to be more numerous in trained mice receiving saline before the reactivation trial than in saline-injected pseudo-trained mice. Injecting propranolol before the reactivation trial prevented the increase in BLA p-ERK positive neurons.

#### Hippocampus

A comparable amount of CA1p-ERK positive neurons was observed across experimental conditions.

## Discussion

We have shown that upon the acquisition of TFC in one context and its subsequent reactivation in another context, mice exhibit intense freezing to the tone alone presented 48 h later in the second context. We then found that this long term fear response is significantly attenuated by pre-reactivation injections of the central/peripheral β-ARs blocker propranolol, but not of the selective peripheral β-ARs blocker sotalol. This observation therefore reveal that attenuation of fear memory results from selective blockade of central β-ARs. Recent data showing that, in healthy subjects, propranolol administrated prior to reactivation of previously learned neutral and emotional material selectively reduces memory for emotional material (Kroes et al., 2010; Schwabe et al., 2013) indicates that the treatment specifically disrupts the storage of emotional information.

Extensive evidence indicates that traumatic memory can be attenuated by administration of β-ARs antagonists at different steps of the consolidation process, i.e., post-trauma, pre-reactivation, or post-reactivation. While the rationale for post-trauma interventions is to prevent the original trace from being consolidated and, hence, to block fear memory formation, propranolol administered before or immediately after fear memory reactivation is expected to interfere with its subsequent reconsolidation (Sara, 2000). Experiments carried out in rodents have, however, predominantly demonstrated the efficacy of post-reactivation treatments (Przybyslawski et al., 1999; Debiec and LeDoux, 2004; Muravieva and Alberini, 2010). To our knowledge, the unique study reporting an effect of pre-reactivation injections of propranolol in the rat show an impairment of inhibitory avoidance memory restricted to the reactivation trial, that was indicative of a state-dependency effect (Muravieva and Alberini, 2010). Our findings therefore provide the first evidence in the mouse that reactivating fear memory under β-ARs blockade attenuates this memory assessed drug free 48 h and, based on the observation post-reactivation β-ARs blockade was ineffective, that this effect is unlikely to be ascribed to a disruptive effect of propranolol memory reconsolidation.

In agreement with previous data (Heinrichs et al., 2013), the number of dendritic spines and, hence, the amount of synaptic inputs on principal BLA spiny neurons was enhanced in conditioned saline-injected mice, confirming that consolidation of an aversive experience durably intensifies the wiring of BLA circuits. Importantly, pre-reactivation i.p. injections of propranolol was found to completely reset the increase in BLA spines, demonstrating that reconsolidating a memory trace under β-ARs blockade interferes with the remodeling of neurons in this region.

The mechanisms by which propranolol blocks TFC-induced spines need, however, to be elucidated. There is evidence that activation of the ERK/MAPK pathway in the amygdala is required for memory consolidation of pavlovian fear conditioning (Huang et al., 2000; Schafe et al., 2000). The β-ARs- (Pu et al., 2009) or stress- (Sarabdjitsingh et al., 2012) dependent facilitation of amygdala LTP, which otherwise promotes the neo-formation of spines (Mitra et al., 2005), is blocked by ERK inhibition (Grissom and Bhatnagar, 2011) suggesting a link between β-ARs, ERK signaling, and spinogenesis in this region. Consistent with this, we first observed that p-ERK levels were increased in saline-injected trained mice and decreased in propranolol-injected trained mice, suggesting that the decrease in TFC memory and the blockade of TFC-induced spines might be ascribed to a propranolol-induced down regulation of p-ERK signaling in BLA neurons. Immunohistochemistry data showing that p-ERK positive neurons were increased in the BLA of saline-injected trained mice compared to both saline-injected pseudo-trained mice and propranolol-injected trained mice provide additional support to this hypothesis. Indeed, the action of p-ERK in the cytoplasm is to enhance protein synthesis by activating downstream signaling pathways. Among those, the mammalian target of rapamycin (mTOR) pathway controls the synthesis of PSD95 and ARC proteins (Ploski et al., 2008) which are implicated in the regulation of the size and shape of spines as well as in the development of dendrite complexity (Kumar et al., 2005). Thus, noradrenergic blockade during reactivation might contribute to the attenuation of traumatic memories by interfering with signaling pathways governing neural and structural plasticity in the BLA.

Surprisingly, although the acquisition of TFC is thought to be hippocampus-independent (Phillips and LeDoux, 1992), the formation of TFC memory was also associated with an increase in spines in CA1 DH neurons. Although unexpected, this finding is nevertheless consistent with the well-characterized neural and cognitive profile of C57BL/6 inbred mice. Specifically, these mice have a highly functional hippocampus (Matsuyama et al., 1997; Nguyen et al., 2000), outperform in tasks requiring spatial and contextual information processing (Ammassari-Teule et al., 2001; Passino et al., 2002) but have difficulties in disentangling elemental stimuli from configural/contextual representations (Restivo et al., 2002). Thus, a possibility exists that, during TFC acquisition, the explicit stimulus -tone- was embedded in a spatial/contextual representation recruiting the DH. Consistent with the independence of DH spines from the formation of emotional representations, no increase in spines was detected in the ventral hippocampus although this region is primarily involved in emotion and stress (Fanselow and Dong, 2010). Moreover, consistent with the independence of DH spines from the activation of the ERK/β-ARs cascade, the increase in DH spines was not accompanied by an increase in p-ERK signaling in the DH region and DH spines were unaffected by propranolol.

Collectively, our results show that peripheral injections of propranolol selectively abolish the increase in dendritic spines and p-ERK signaling in the BLA while its effect on the fear response is less pronounced. These observations raise a point of increasing relevance to fear memory studies, i.e., how individual differences in the capacity or in the organization of memory interfere with the propensity to show fear symptoms. For example, in humans, the genetic predisposition to build up strong memories increases the risk of developing PTSD after a traumatic event (de Quervain et al., 2012). Our data suggest that, in the same fashion, specific stimulus processing modalities, as the predisposition to include the tone in a contextual/configural representation, and therefore to engage the hippocampus in a task in which it is dispensable, might lead to the formation of a multifaceted memory trace (i) enhancing connectivity at multiple brain sites and (ii) decreasing the sensitivity of the trace to treatments selectively targeting β-ARs in the BLA.

## Author contributions

Martine Ammassari-Teule and Gisella Vetere designed, directed and coordinated the study. Gisella Vetere, Valentina Piserchia, Giovanni Novembre, and Massimiliano Aceti conducted behavioral experiments. Gisella Vetere and Valentina Piserchia, performed spine analysis. Gisella Vetere and Antonella Borreca performed western blotting analysis. Gisella Vetere performed statistical analysis. Martine Ammassari-Teule wrote the manuscript, with assistance from Gisella Vetere and Antonella Borreca.

## Grants

This work was supported by a grant from the Italian Ministry of Health (progetto finalizzato N° RF09.263) to Martine Ammassari-Teule. These data were presented by Gisella Vetere as an oral communication at the European Science Foundation Conference on the Dynamic Brain (“Neurobiology of Emotions, Stresa, 11–14 November 2012).

## Conflict of interest statement

The authors declare that the research was conducted in the absence of any commercial or financial relationships that could be construed as a potential conflict of interest.

## Supplementary material

Figure S1 **Sotalol does not affect freezing during fear reactivation and fear memory testing**. Injecting the selective peripheral β-adrenergic receptor blocker sotalol before the reactivation trial does not reduce freezing during the reactivation trial (left panel) and the long term memory test (right panel) run drug free 48 h later. Data are expressed as mean ± s.e.m.

Figure S2 **Propranolol injected post-reactivation does not reduce fear memory**. As already shown in Figure 1, mice injected with propranolol before the reactivation session showed significantly less freezing during the memory test. However, propranolol injected immediately after the reactivation trial does not significantly reduce freezing during the long term memory test run drug free 48 h later (5 mice per group). Data are expressed as mean ± s.e.m. ^*^*p* < 0.025.

Figure S3 **Fear memory does not promote spine growth in the ventral hippocampus**. Mice subjected to tone fear conditioning and injected with saline before the reactivation trial did not show any increase in spine density in ventral hippocampus CA1 neurons upon the long term memory test. Data are expressed as mean ± s.e.m.

Figure S4 **Propranolol prevents the increase in p-ERK levels observed upon fear memory testing in the BLA**. Immunohistochemistry staining confirmed western blotting analyses. Mice trained for tone fear conditioning and injected with saline before the reactivation trial showed an increase in BLA p-ERK positive neurons in comparison with saline-injected pseudo-trained mice. Trained mice injected with propranolol before the retention trial showed a consistent reduction in p-ERK positive neurons in the BLA compared to their saline-injected trained counterpart (upper panels). In the CA1 field of the hippocampus, the amount of p-ERK positive neurons did not show variations across experimental conditions. (lower panels).
