# Supplementary material for: Reactivating fear memory under propranolol resets pre-trauma levels of dendritic spines in basolateral amygdala but not dorsal hippocampus neurons
Source: Front Behav Neurosci. 2013 Dec 23;7:211. doi: 10.3389/fnbeh.2013.00211 (PMC3870275; doi:10.3389/fnbeh.2013.00211)
Supplement: Figure S1 — Sotalol does not affect freezing during fear reactivation and fear memory testing. Injecting the selective peripheral β-adrenergic receptor blocker sotalol before the reactivation trial does not reduce freezing during the reactivation trial (left panel) and the long term memory test (right panel) run drug free 48 h later. Data are expressed as mean ± s.e.m. [file Presentation1.PPT]

## Slide 1
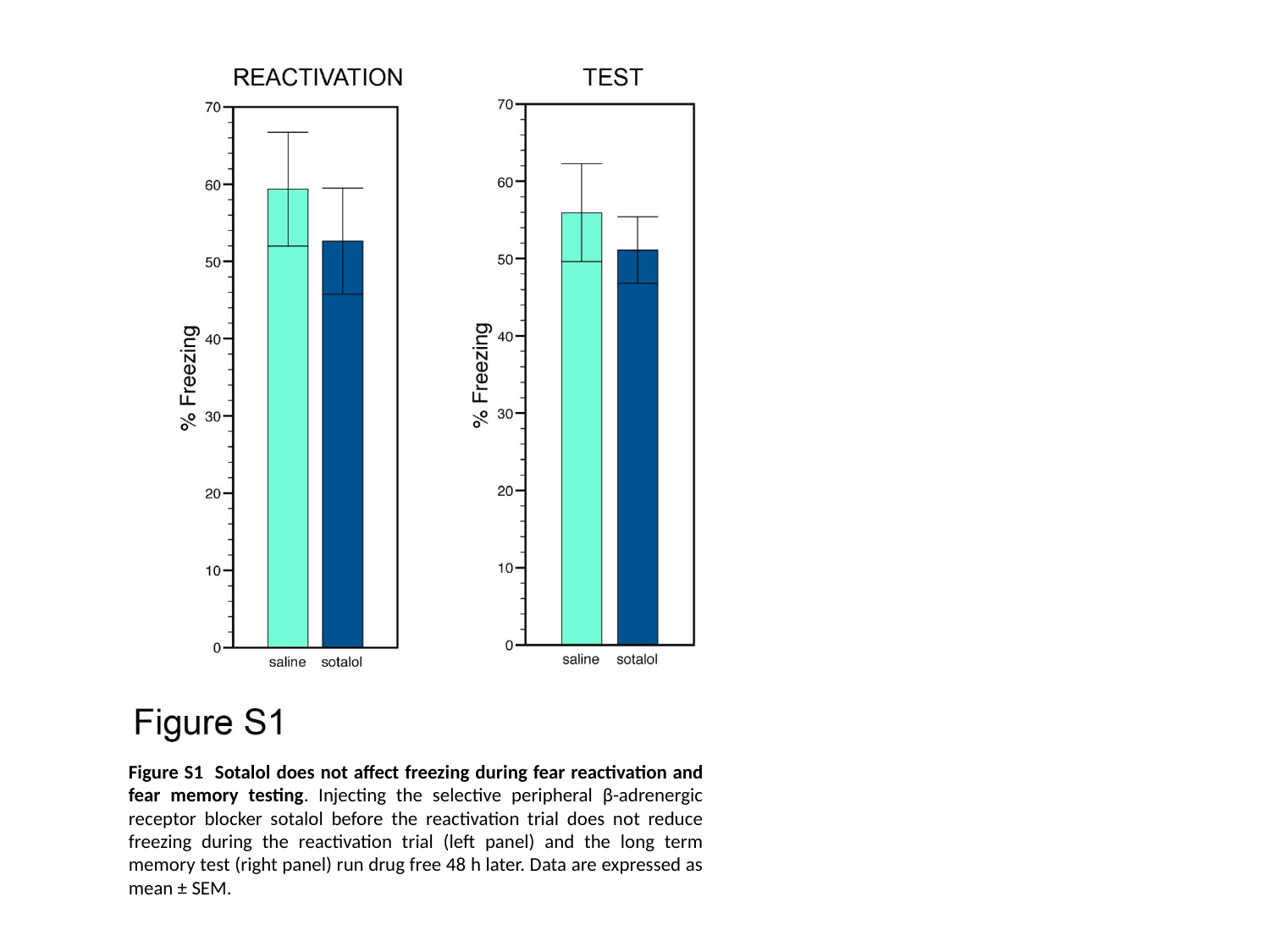

Figure S1 Sotalol does not affect freezing during fear reactivation and fear memory testing. Injecting the selective peripheral β-adrenergic receptor blocker sotalol before the reactivation trial does not reduce freezing during the reactivation trial (left panel) and the long term memory test (right panel) run drug free 48 h later. Data are expressed as mean ± SEM.

## Slide 2
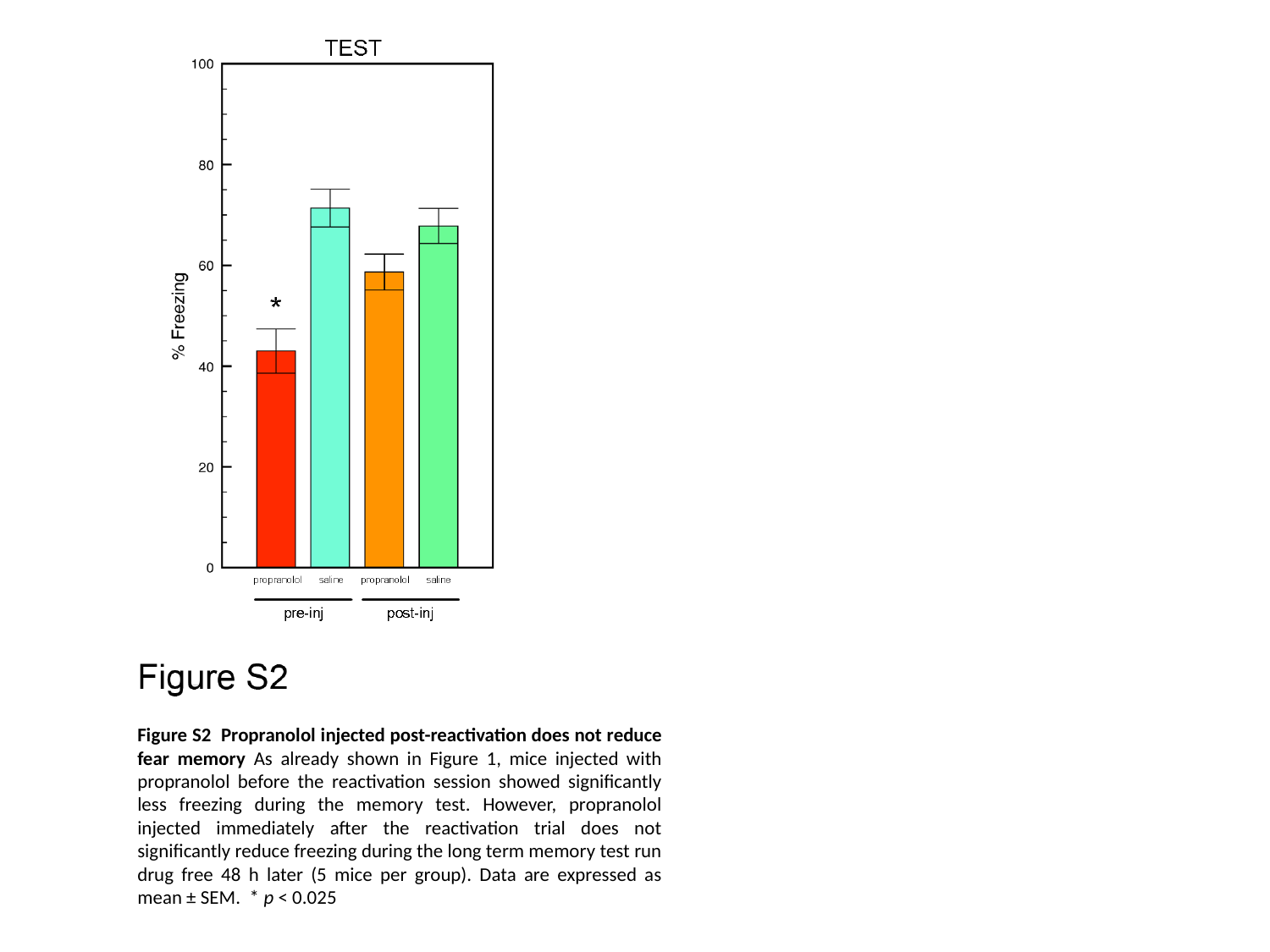

Figure S2 Propranolol injected post-reactivation does not reduce fear memory As already shown in Figure 1, mice injected with propranolol before the reactivation session showed significantly less freezing during the memory test. However, propranolol injected immediately after the reactivation trial does not significantly reduce freezing during the long term memory test run drug free 48 h later (5 mice per group). Data are expressed as mean ± SEM. * p < 0.025

## Slide 3
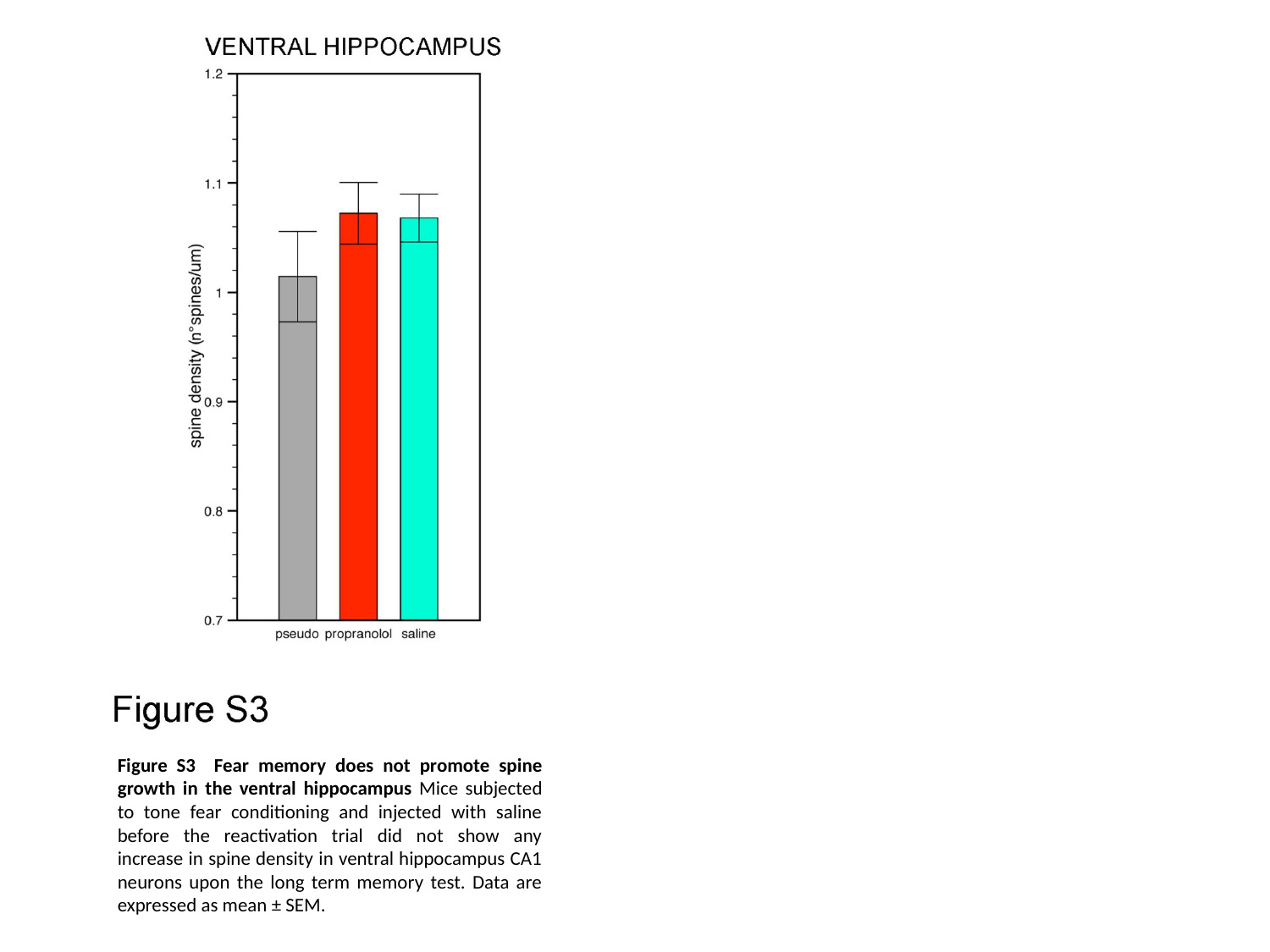

Figure S3 Fear memory does not promote spine growth in the ventral hippocampus Mice subjected to tone fear conditioning and injected with saline before the reactivation trial did not show any increase in spine density in ventral hippocampus CA1 neurons upon the long term memory test. Data are expressed as mean ± SEM.

## Slide 4
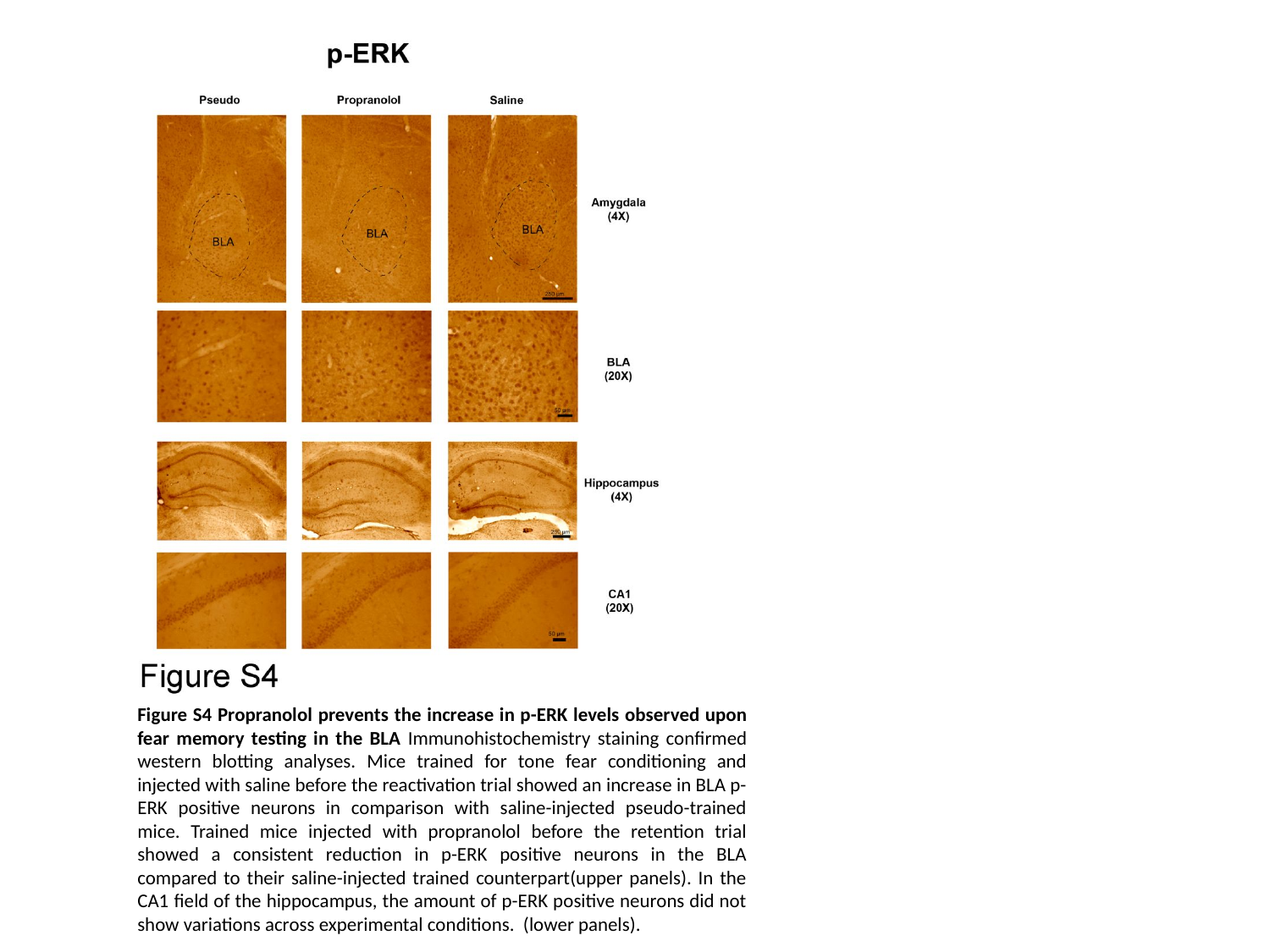

Figure S4 Propranolol prevents the increase in p-ERK levels observed upon fear memory testing in the BLA Immunohistochemistry staining confirmed western blotting analyses. Mice trained for tone fear conditioning and injected with saline before the reactivation trial showed an increase in BLA p-ERK positive neurons in comparison with saline-injected pseudo-trained mice. Trained mice injected with propranolol before the retention trial showed a consistent reduction in p-ERK positive neurons in the BLA compared to their saline-injected trained counterpart(upper panels). In the CA1 field of the hippocampus, the amount of p-ERK positive neurons did not show variations across experimental conditions. (lower panels).
